# Comment on Hopkins et al. Value of the Lung Immune Prognostic Index in Patients with Non-Small Cell Lung Cancer Initiating First-Line Atezolizumab Combination Therapy: Subgroup Analysis of the IMPOWER150 Trial. *Cancers* 2021, *13*, 1176

**DOI:** 10.3390/cancers13143624

**Published:** 2021-07-20

**Authors:** Edouard Auclin, Laura Mezquita, Benjamin Besse

**Affiliations:** 1 Department of Medical and Thoracic Oncology, Hôpital Européen Georges Pompidou, Assistance Publique des Hôpitaux de Paris, Université de Paris, 75015 Paris, France; edouard.auclin@aphp.fr; 2 Centre d’Investifation Clinique (Epidémiologie Clinique) INSERM 1418, Hôpital Européen Georges Pompidou, Assistance Publique des Hôpitaux de Paris, 75015 Paris, France; 3 Medical Oncology Department, Hospital Clinic of Barcelona, 08036 Barcelona, Spain; 4 Laboratory of Translational Genomics and Targeted Therapies in Solid Tumors, The August Pi i Sunyer Biomedical Research Institute IDIBAPS, 08036 Barcelona, Spain; 5 Department of Oncology, Institut Gustave Roussy, 94805 Villejuif, France; benjamin.besse@gustaveroussy.fr

We read, with great interest, the recently published article by Hopkins et al. reporting for the first time the prognostic value of the lung immune prognostic index (LIPI) in a cohort of patients treated in a clinical trial with atezolizumab for advanced non-small cell lung cancer (NSCLC) [1]. In their post hoc analysis of the IMpower 150 trial, the authors included patients treated in the three treatment arms, atezolizumab-carboplatin-paclitaxel (ACP, *n =* 382), bevacizumab-carboplatin-paclitaxel (BCP, *n =* 381), and atezolizumab-bevacizumab-carboplatin-paclitaxel (ABCP, *n =* 385).

The LIPI correlated with survival outcomes in patients receiving atezolizumab with or without the addition of bevacizumab. However, the authors did not show the results for the treatment arm without atezolizumab (BCP). The magnitude of benefit with the addition of atezolizumab differed between the LIPI groups. Notably, in the poor LIPI group no benefit was observed with the addition of atezolizumab.

We congratulate the authors for this interesting and important study. Here, we provide an overview of the impact of the LIPI and host-related inflammatory biomarkers on immunotherapy outcomes, and address some issues raised by the study of Hopkins et al.

## 1. LIPI Is a Strong Prognostic Factor in Both Patients Treated with Immunotherapy in Combination with Chemotherapy, and also in Treatment-Naive Patients

LIPI’s prognostic value for survival outcomes in NSCLC treated with immune checkpoint inhibitors (ICIs) was first published in 2018 [2]. At that time, our group performed a retrospective study in patients treated with immunotherapy in the second-line setting or beyond. In a validation cohort of patients treated with chemotherapy only (i.e., immunotherapy naive), there was no correlation with outcome, suggesting the hypothesis that LIPI may be predictive for outcome with immunotherapy. In a subsequent and larger retrospective study, Kazandjian et al. validated the LIPI’s prognostic value in patients included in prospective trials who were treated with immunotherapy, chemotherapy, and targeted therapy [3]. Although LIPI was prognostic regardless of the treatment used, its discriminatory capacity appeared greater in patients treated with immunotherapy. Then, in 2019, the Hopkin’s group demonstrated the value of the LIPI for patient risk stratification under atezolizumab as monotherapy [4].

In the context of combination therapy of chemotherapy accompanied with immunotherapy (and anti-angiogenics), this represents the first study to validate the LIPI in treatment-naïve patients. Our team recently reported similar results in a preliminary analysis of patient cohorts treated with immunotherapy and chemotherapy or an immunotherapy and immunotherapy association in the first-line setting [5].

Taken together, these studies (including Hopkins et al.) highlight the importance of host-related inflammatory biomarkers as a prognostic factor for outcomes in patients treated with immunotherapy for NSCLC.

## 2. Does LIPI Offer Additional Guidance for Treatment Selection in NSCLC Patients?

Today, there is no doubt that LIPI is a prognostic marker not only for lung cancer, but also for other solid tumors. However, its predictive value has not yet been proven. In light of previously published data by the Hopkins’ group, new data are provided supporting that LIPI could guide treatment decisions. Firstly, the authors had already suggested that LIPI may be predictive for atezolizumab efficacy in a monotherapy setting [4]. Patients with poor LIPI did not derive benefits from immunotherapy compared with docetaxel. The most recent study by Hopkins et al. speculated the same interesting predictive value of LIPI in a combination setting, with patients with poor LIPI having no survival advantage (OS and PFS) with the addition of atezolizumab to chemotherapy and bevacizumab. However, the interaction term was not significant for OS (*p* = 0.66) and there was a trend to significance for PFS (*p* = 0.13). This could be explained by a lack power (*n =* 121 in the poor LIPI group).

Along the same lines, in the study by Kazandjian et al., the poor LIPI group seemed to have poorer OS and PFS when treated with immunotherapy compared with chemotherapy alone [3]. Unfortunately, in this latter study, the authors did not explore the predictive value of LIPI statistically.

We believe that this aspect merits further exploration in the randomized clinical trial setting with different treatment combinations and with a focus on the poor LIPI population as, in fact, we have shown that these patients can respond well to ICI combinations [5]. The combination of tumor mutational burden (TMB) with circulating inflammatory biomarkers could be an interesting path toward patients’ selection. Although TMB calculation is not well standardized and the genes responsible for ICI sensitivity need to be identified more clearly, it seems that TMB could be another interesting predictive biomarker for ICI response [6].

## 3. LIPI Is an Agnostic Prognostic Biomarker

The prognostic value of the LIPI score was already shown not only in several studies in NSCLC but also in other tumor types treated with immunotherapy-containing regimens [7,8,9]. This confirms that the prognostic impact of LIPI is not restricted to patients with lung cancer, and that it can be considered a pan-tumor biomarker mainly related to the immune context of the patient rather than to the tumor. We have systematically shown in many cohorts of different solid tumors that the consideration of the host inflammatory biomarkers is a major prognostic aspect under ICIs and may play a role in treatment tailoring for cancer patients. For example, we recently presented data from a cohort of 151 patients treated with ICIs for microsatellite instable (MSI-H) tumors [10]. In this theoretically ‘good responder’ population, patients with poor LIPI had very poor outcomes, highlighting the important role that host-related biomarkers can play in predicting the ICI response.

## 4. How Can We Implement Host-Related Inflammatory Biomarkers into Routine Clinical Practice?

Host-related inflammatory markers are not currently routinely included when stratifying patients in clinical trials. Yet, as highlighted above, they are highly prognostic for survival and response to immunotherapy in patients with cancer, regardless of the presumed drug efficacy. Host-related inflammatory markers could impact the results of clinical trials. Unbalanced LIPI groups could lead to an “immunotherapy arm” enriched in poor LIPI patients, and thus, artificially decrease the apparent treatment effect in this arm.

LIPI is a simple and accessible scoring system. All components of this score (i.e., leucocyte and neutrophil counts, lactate dehydrogenase) are mandatory data in the majority of clinical trials and LIPI analysis would provide additional information to better stratify the population in further randomized studies.

We propose the following approach to address the question of how to integrate these markers into routine clinical practice:

1st step: validate LIPI retrospectively in previous clinical trials with immunotherapy (*post-hoc* analyses of the databases from princeps immunotherapy trials in Oncology)

2nd step: design prospective clinical trials including LIPI as a stratification factor

3rd step: design prospective clinical trials using LIPI as a marker for guiding treatment selection

In the Hopkins et al. study, the LIPI groups were well balanced between the treatment arms. The results concerning the prognostic and predictive values of LIPI are particularly interesting and should garner enthusiasm of the scientific community to further explore host-related inflammatory biomarkers.

## 5. Data from This Study We Would Have Hoped to See

IMpower 150 is the only study to date to show a positive impact of immunotherapy in an *EGFR*/*ALK* population. We are surprised the analysis of the LIPI amongst patients with a driver mutation was not reported in the current paper. Kazandjian et al. reported that LIPI was also prognostic in patients treated with anti-*EGFR* and anti-*ALK* targeted therapy. It would be of particular value to see if LIPI has the same impact in the context of chemotherapy with atezolizumab and bevacizumab. As mentioned above, LIPI could be interesting in selecting patients for these kinds of combination therapies.

The LIPI identifies a population of patients with a poor prognosis under ICI (poor LIPI group), namely a short PFS and OS and high rate of early death (defined as OS < 3 months). This poor LIPI population does not derive any benefit from ICI in the first-line setting or beyond. We think that the results of Hopkins et al. would gain in impact from showing the early death rate in the treatment arms according to the LIPI groups.

Finally, in the IMpower 150 study, no cross-over to atezolizumab was permitted in cases of progression. However, some patients may have received immunotherapy as second-line treatment in the routine care setting. This crossover can impact OS, as we previously showed that LIPI was prognostic in the second-line setting and beyond. We would be very interested if the authors could show these data for patients who did not crossover in the chemotherapy plus bevacizumab arm.

## 6. Integrating Host-Related Inflammatory Markers in NSCLC will Improve the Selection of Candidates for Each Therapy

The work of Hopkins et al., together with that of our and other groups, support the importance of host-related inflammatory biomarkers represented by the LIPI on outcomes under immunotherapy in NSCLC. To date, only tumor-related biomarkers are taken into account when choosing the patient’s treatment, with PD-L1 status being the most commonly used marker. As previously shown, the LIPI is independently associated with both survival and response under immunotherapy [5]. We consider it to be of very great importance to integrate host-related inflammatory markers into the algorithm containing tumor-related biomarkers in order to improve patient selection for immunotherapy therapeutic strategies for both monotherapy and combination therapy in NSCLC.

We would be interested in hearing the perspective of the Hopkin’s team and others on the points raised in this commentary.
